# Phenyl-substituted aminomethylene-bisphosphonates inhibit human P5C reductase and show antiproliferative activity against proline-hyperproducing tumour cells

**DOI:** 10.1080/14756366.2021.1919890

**Published:** 2021-06-09

**Authors:** Giuseppe Forlani, Giuseppe Sabbioni, Daniele Ragno, Davide Petrollino, Monica Borgatti

**Affiliations:** a Department of Life Science and Biotechnology, University of Ferrara, Ferrara, Italy; b Department of Chemical, Pharmaceutical and Agricultural Sciences, University of Ferrara, Ferrara, Italy

**Keywords:** Antiproliferative activity, P5C reductase inhibitors, proline-P5C cycle, proline-overproducing tumours

## Abstract

In certain cancers, such as breast, prostate and some lung and skin cancers, the gene for the enzyme catalysing the second and last step in proline synthesis, δ^1^-pyrroline-5-carboxylate (P5C) reductase, has been found upregulated. This leads to a higher proline content that exacerbates the effects of the so-called proline-P5C cycle, with tumour cells effectively using this method to increase cell survival. If a method of reducing or inhibiting P5C reductase could be discovered, it would provide new means of treating cancer. To address this point, the effect of some phenyl-substituted derivatives of aminomethylene-bisphosphonic acid, previously found to interfere with the catalytic activity of plant and bacterial P5C reductases, was evaluated *in vitro* on the human isoform 1 (PYCR1), expressed in *E. coli* and affinity purified. The 3.5-dibromophenyl- and 3.5-dichlorophenyl-derivatives showed a remarkable effectiveness, with IC_50_ values lower than 1 µM and a mechanism of competitive type against both P5C and NADPH. The actual occurrence *in vivo* of enzyme inhibition was assessed on myelogenous erythroleukemic K562 and epithelial breast cancer MDA-MB-231 cell lines, whose growth was progressively impaired by concentrations of the dibromo derivative ranging from 10^−6^ to 10^−4 ^M. Interestingly, growth inhibition was not relieved by the exogenous supply of proline, suggesting that the effect relies on the interference with the proline-P5C cycle, and not on proline starvation.

## Introduction

**Figure 1.**
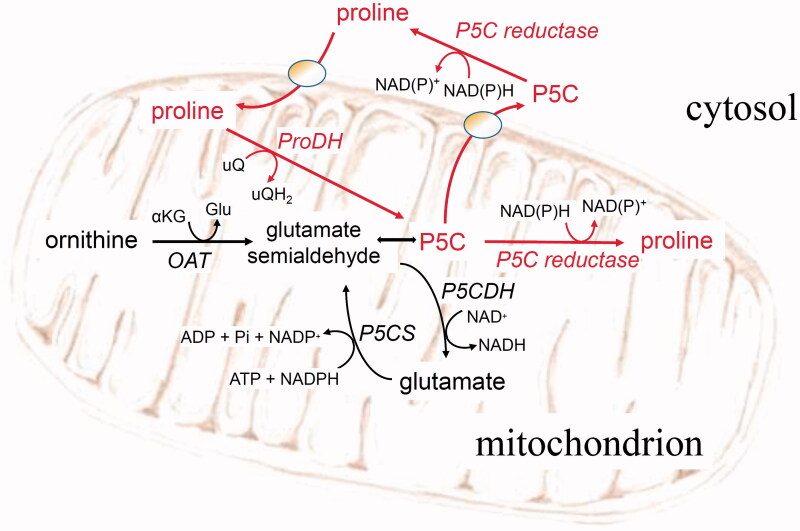
The proline-P5C cycle. Abbreviations that are not listed in the text: OAT: ornithine-δ-aminotransferase; αKG: α-ketoglutarate; Glu: glutamate; P5CS: P5C synthetase; uQ: oxidised ubiquinone; uQH_2_: reduced ubiquinone.

Tumour development implies a deep reprogramming of cell metabolism, often inducing some additional or differential metabolic dependencies. These can be exploited for the identification of new therapeutic strategies based on substances able to target specific requirements of malignant cells^1^. Among the changes in the metabolic fluxes undergone by tumoral cells to satisfy a larger demand for carbon skeletons, ATP and reducing power, increasing rates of the so-called proline-P5C cycle seem to play a significant role in several cancer types^2–3^. Although some other mechanisms have been also hypothesised, such as an increase of collagen synthesis and maturation and the acquisition of cancer cell plasticity and heterogeneity^4^, the main contribution of proline metabolism to tumorigenesis seems to rely upon the consequently augmented redox cycling and maintenance of pyridine nucleotides^5^. Proline is synthesised from glutamate or ornithine in short pathways sharing the last step, the NAD(P)H-dependent reduction of the common intermediate δ^1^-pyrroline-5-carboxylic acid (P5C) by P5C reductase (EC 1.5.1.2)^6^. P5C is also formed during the mitochondrial degradation of proline to glutamate, which involves two oxidative steps catalysed in sequence by proline dehydrogenase (ProDH; EC 1.5.5.2)^7^ and P5C dehydrogenase (P5CDH, also known as aldehyde dehydrogenase 4; EC 1.2.1.88)^8^. The former is believed to feed electrons directly to the respiratory ubiquinone pool,^9^ whereas the latter uses NAD^+^ as the electron acceptor^10^. The occurrence of a shortcut in which the P5C released by ProDH is not further oxidised by P5CDH, but is reduced back to proline by P5C reductase, has been early hypothesized^11^. Such apparently futile proline-P5C cycle (Figure 1) may provide the cell with a mechanism for transferring reducing equivalents from the cytosol to the mitochondrion^11–12^, and to fuel the respiratory chain^13^. Moreover, ProDH activity may alternatively lead to ROS production^14^, which can trigger in turn the apoptotic mechanism^15^, or increased ATP synthesis for protective autophagy^16^. Although in plants the occurrence of the proline-P5C cycle is still a matter of debate due to the physical separation of P5C reductase and ProDH^17^, its role in human cell is now well established^18^. Consistently, high levels of expression of both ProDH and P5C reductase have been reported in a series of cases in which cell metabolism needs to be enhanced, for instance during nutrient stress^19^ or metastasis formation^20^.

Being highly expressed in several cancer types and possibly linked to tumour cell survival^2–4^^,^^21–23^, the activity of P5C reductase represents a potential target for the development of new therapeutic approaches^24^. In fact, recent studies on breast^25^ and prostate^26^ cancer cell lines demonstrated that P5C reductase knockout was effective in inducing a reduction in tumour size *in vivo* and an increase in cell cycle arrest and apoptosis *in vitro*, respectively. These results would open interesting perspectives through gene therapy, when approved. In the meantime, an amenable approach would be represented by the identification of specific and effective inhibitors of P5C reductase, to be used as antiproliferative agents. A previous screening of a commercially available library of pharmaceutically active compounds pointed out pargylin as a fragment-like hit, with a modest IC_50_ of 200 μM, but an encouraging ligand efficiency. The analysis of a number of analogues allowed identifying 4-bromopargyline as the most effective inhibitor, showing an IC_50_ value of 9 μM. However, the treatment of cancer cells with the compound resulted in mild effects, with 30–40% reduction of breast cancer cell proliferation at 10 μM, and an unclear dose-activity relationship on intracellular free proline at higher levels^27^.

Inhibitors of P5C reductase have been previously exploited as potential herbicides. A preliminary screening *in vitro* pointed out the ability of some aminomethylene bisphosphonic acids to interfere with the activity of the plant enzyme in the micromolar to millimolar range^28^. Synthesis and analysis of several active derivatives allowed defining the steric and electronic requirements for maintenance or enhancement of the inhibitory properties^29^, and identifying 3.5-dibromophenyl-aminomethylene bisphosphonic acid as the most effective analogue^30^. The ability of the latter to inhibit plant growth, and cause the accumulation of P5C levels in treated seedlings, proved that the inhibition of P5C reductase takes place *in vivo*^31^. Moreover, the same compounds were found even more active *in vitro* against P5C reductase of the human pathogen *Streptococcus pyogenes*, although concentrations 2 orders of magnitude higher than IC_50_ were required to inhibit bacterial growth^32^. Based on this rationale, we investigated the effectiveness of aminomethylene-bisphosphonic acids in inhibiting human P5C reductase and reducing proliferation of some selected tumour cells lines.

## Methods

### Substrates, reagents and inhibitors

Unless specified otherwise, all compounds were purchased from Sigma-Aldrich, and were of analytical grade. Δ^1^-pyrroline-5-carboxylic acid was synthesised by the periodate oxidation of δ-*allo*-hydroxylysine (Sigma H0377) and purified by cation-exchange chromatography onto a Dowex AG50W-X4 (200–400 mesh) column, as described^33^. DL-P5C solutions in 1 M HCl were stored at 4 °C in the dark, and brought to neutral pH just before the assay using proper aliquots of a 1 M Tris base solution. Pargyline (*N*-methyl-*N*-(2-propynyl)benzylamine hydrochloride) was from Sigma (P8013), whereas 4-bromopargyline^27^ was a generous gift from prof. Reuven Agami (The Netherlands Cancer Institute, Amsterdam, The Netherlands). Phenyl-substituted aminomethylene-bisphoshonic acids were synthesised as described^29^. For 3,5-dibromophenylaminomethylene-bisphosphonic acid (Br_2_PAMBPA) a slightly different procedure was adopted, as it follows.

### Procedure for the synthesis of 3,5-dibromophenylaminomethylene-bisphosphonic acid

3,5-Dibromoaniline (3.01 g, 12.00 mmol), triethyl orthoformate (2.40 mL, 14.40 mmol) and diethyl phosphite (6.18 mL, 48.00 mmol) were vigorously stirred and heated at 120 °C for 16 h. After cooling, volatiles were evaporated under reduced pressure. Trituration of the solid with n-hexane followed by filtration furnished the pure ester tetraethyl (3,5-dibromophenylaminomethylene) bisphosphonate (4.38 g, 68% yield) which was further dissolved in concentrated hydrochloric acid (300 mL) and refluxed for 4 h. After this period the reaction mixture was cooled and concentrated under reduced pressure affording a solid which was washed with small amounts of cold distilled water and dried under vacuum to obtain the pure bisphosphonic acid Br_2_PAMBPA as a whitish solid (3.31 g, 65% overall yield). For NMR analysis ^1^H (300 MHz), ^13 ^C (101 MHz), ^31 ^P (122 MHz) NMR spectra (Supplementary Figures S1 and S2) were recorded in CDCl_3_ or DMSO-*d_6_* solutions at room temperature. The chemical shifts in ^1^H and ^13 ^C NMR spectra were referenced to trimethylsilane (TMS), while in ^31 ^P NMR were referenced to 85% H_3_PO_4_ in D_2_O. Peak assignments were aided by ^1^H-^1^H COSY and gradient-HMQC experiments.

*Tetraethyl (3,5-Dibromophenylaminomethylene)bisphosphonate*: ^1^H NMR (300 MHz, CDCl_3_) *δ* = 7.04 (s, 1H, Ar), 6.77 (s, 2H, Ar), 4.35 (bs, 1H, NH), 4.27–4.08 (m, 8H, OCH_2_), 4.05 (t, ^2^*J*_H-_*_P_* = 22.0 Hz, 1H, CHP2), 1.32 (t, ^1^*J*_H-H_ = 7.1 Hz, 6H, CH_3(a)_), 1.28 (t, ^1^*J*_H-H_ = 7.1 Hz, 6H, CH_3(b)_); ^13 ^C NMR (101 MHz, CDCl_3_) *δ* = 148.2 (C, Ar), 124.2 (CH, Ar), 123.4 (2 C, Ar), 115.2 (2 CH, Ar), 63.9 (2 OCH_2(a)_), 63.6 (2 OCH_2(b)_), 49.9 (t, ^1^*J*_C-_*_P_* = 148.0 Hz, CHP2), 16.4 (2 CH_3(a)_), 16.3 (2 CH_3(b)_); ^31 ^P NMR (122 MHz, CDCl_3_) *δ* = 16.8 (2 P).

*3,5-Dibromophenylaminomethylenebisphosphonic acid*: ^1^H NMR (300 MHz, DMSO-*d_6_*) *δ* = 9.88 (bs, 4H, 4 OH), 6.97 (s, 2H, Ar), 6.81 (s, 1H, Ar), 6.18 (bs, 1H, NH), 3.96 (d, ^2 ^*J* = 22.0 Hz, CHP_2_); ^13 ^C NMR (101 MHz, DMSO-*d_6_*) *δ* = 151.2 (C, Ar), 122.8 (CH, Ar), 120.3 (2 C, Ar), 114.8 (2 CH, Ar), 50.8 (t, ^1^*J*_C-_*_P_* = 140.0 Hz, CHP_2_); ^31 ^P NMR (122 MHz, DMSO-*d_6_*) *δ* = 15.0 (2 P).

### Purification of human P5C reductase

The gene coding for P5C reductase 1 (*PYCR1*, gene ID 5831), cloned into the expression vector pET28a^+^^34^, was kindly provided by prof. Zihe Rao (Tsinghua University, China). For heterologous expression, *E. coli* BL21(DE3) pLysS cells (Invitrogen) were made competent by the calcium chloride method, transformed with the recombinant vector and selected on kanamycin-containing LB plates. About 6 h after inducing gene expression in liquid LB medium at 24 °C by the addition of 1 mM isopropyl-D-thiogalactopyranoside, cells were harvested by centrifugation and stored at −20 °C. Aliquots (about 1 g cell material) were lysed in an ice-cold mortar with 2 g g^−1^ alumina and resuspended in 20 mL g^−1^ extraction buffer (50 mM Na phosphate buffer, pH 7.5, containing 200 mM NaCl and 20 mM imidazole). The His-tagged protein was purified from clarified extracts by affinity chromatography onto a His-Trap^TM^ FF column (1 mL, GE Healthcare 17–5319-01). Stepwise elution was achieved by increasing concentrations of imidazole in extraction buffer. Discontinuous SDS–polyacrylamide gel electrophoresis was performed at 8 mA with a 5% stacking and a 12% separating gel, using a Minigel system (BioRad). Samples were mixed with the same volume of 125 mM Tris–HCl buffer (pH 6.8) containing 4% (w/v) SDS, 20% (v/v) glycerol and 10% (v/v) β-mercaptoethanol, and denatured 5 min at 100 °C. Proteins were visualised by soaking gels in Quick Coomassie Stain (CliniSciences) overnight.

### P5C reductase assay

Enzyme activity was measured at 30 °C as the P5C-dependent oxidation of NAD(P)H. Assays were performed in 96-microwell plates in a final volume of 0.2 mL. The assay mixture contained 4 mM DL-P5C and 0.5 mM of either NADH or NADPH in 50 mM Tris-HCl buffer, pH 7.5. Parallel blanks were performed in which P5C had been omitted. The decrease of absorbance was measured for 5 min at 0.5-min intervals using a Ledetect plate reader (Labexim, Lengau, Austria) equipped with a LED plugin at 340 nm. Residual NAD(P)H content was estimated on the basis of calibration curves obtained under the same conditions. Activity was calculated by linear regression of data using Prism 6 for Windows, version 6.03 (GraphPad Software, San Diego, CA); one unit of activity was defined as the amount of enzyme that catalyses the P5C-dependent oxidation of 1 nmol NAD(P)H s^−1^ (nkat). Proteins were measured by the Coomassie Brilliant Blue method^35^, using bovine serum albumin as the standard. To evaluate substrate affinity, invariable substrates were fixed at the same levels as in the standard assay. For variable substrates, L-P5C ranged from 400 to 2000 μM, while the concentration of NADH and NADPH ranged from 125 to 500 μM. To evaluate the inhibition brought about by pargylines and aminomethylene-bisphosphonates, freshly prepared 10 mM stock solution in water and 100 mM Tris-HCl buffer pH 7.5, respectively, were diluted with water and added to the reaction mixture before enzyme addition. Parallel control samples were carried out in the absence of any inhibitor. Activity was calculated as described above. Mean activity value in controls was set as 100%, and activity in treated samples was expressed as percent of such value. Percent values were plotted against the logarithm of inhibitor concentration, and data were interpolated using the equation *Y* = 100/(1 + 10^∧^((LogIC_50_-*X*)*HillSlope)). Assays were performed in triplicate (technical replications). Each experiment was repeated at least twice with different enzyme preparations (biological replicates). *K_M_* and *V*_max_ values, as well as the concentrations causing 50% inhibition (IC_50_), *K_I_* values and their confidence intervals were estimated by non-linear regression analysis using Prism 6.

### Human cell lines and growth conditions

Human myelogenous erythroleukemic K562 cells^36^ were cultured in a humidified atmosphere of 5% CO_2_/air either in complete RPMI 1640 medium (Lonza, Verviers, Belgium) or in a modified medium without L-proline (United States Biological, Massachusetts, USA), both supplemented with 10% foetal bovine serum (FBS; Biowest, Nuaillé, France), 100 U mL^−1^ penicillin and 100 μg mL^−1^ streptomycin. Human epithelial breast cancer MDA-MB-231 cells^37^ were cultured under the same conditions in DMEM medium (Lonza, Verviers, Belgium) supplemented with 10% FBS, 100 U mL^−1^ penicillin, 100 μg mL^−1^ streptomycin and 2 mM L-glutamine.

### Antiproliferative assays

K562 cells in the late exponential phase of growth were seeded in 1 mL medium without FBS in 24-well plates to an initial density of about 20,000 cells mL^−1^. Treatments with Br_2_PAMBPA in the range 1–200 µM were carried out 5 h after seeding. Cell growth was monitored at increasing time after the treatment by determining cell density on 50-μL aliquots with a Z1 Coulter Counter (Coulter Electronics), considering a dimensional range between 8 and 20 μm. MDA-MB-231 cells in the late exponential phase of growth (90% confluence) were washed with sterile PBS, incubated with sterile trypsin-EDTA solution (Sigma-Aldrich, Saint Louis, USA) for 2 min, resuspended in fresh DMEM medium and centrifuged at 1000 *g* at room temperature for 5 min. Pellets were resuspended in sterile PBS and spun again to remove any residue of trypsin. Cells were finally resuspended in DMEM medium without FBS, and seeded in 24-well plates in a final volume of 1 mL to an initial density of about 20,000 cells mL^−1^. Treatments with Br_2_PAMBPA in the range 1–200 µM were carried out 5 h after seeding, and 2% FBS was added to each well 24 h thereafter. Cell growth was monitored at increasing time after the treatment by destructive harvest. For both cell lines each treatment was carried out at least in triplication, and each experiment was performed at least twice.

### Cell viability assay

Cell viability was assessed by the Trypan Blue exclusion test^38^. Stained cells were counted in a Bürker chamber. Viable and nonviable cells were recorded separately, and the results of three independent counts were pooled. Data were expressed as percent of alive cells.

### Measurement of intracellular free proline and P5C levels

For proline and P5C quantification, treatments were carried out in T-25 flasks containing 10 mL medium. Cells were harvested by centrifugation at 1000 *g* for 5 min at RT. Each pellet was resuspended in 1.4 mL of sterile PBS and spun again to remove the medium. Washed pellets were transferred on ice, resuspended in a minimal volume of a 3% (w/v) solution of 5-sulfosalicylic acid and subjected to various freeze-thaw cycles to induce rupture of the phospholipid membrane. Cell debris were sedimented by centrifugation at 12,000 *g* for 5 min at RT, and the supernatants were analysed by the acid ninhydrin method of Williams and Frank, as described^33^.

## Results

### Effect of phenyl-substituted aminomethylenebisphosphonates on the activity of human P5C reductase 1

**Figure 2.**
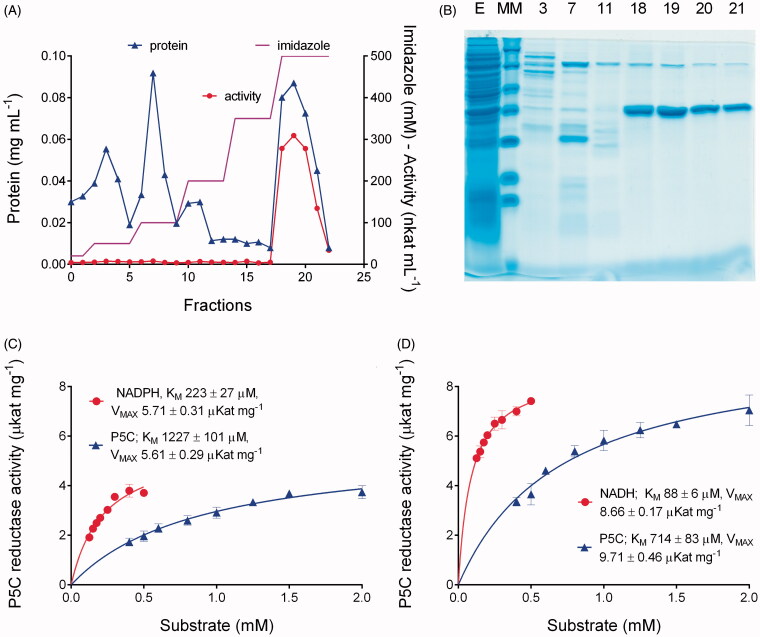
Purification and kinetic characterisation of human P5C reductase. The gene for P5C reductase 1 (*PYCR1*, gene ID 5831) was heterologously expressed in *E. coli*, and the recombinant His-tagged protein was affinity purified by a stepwise gradient of imidazole (panel A). Homogeneity of purified preparations was checked by SDS-PAGE (panel B). Number of lanes in the gel refer to the fractions in panel A; E, crude extract; MM, molecular markers (Fermentas SM0431). Specific activity of the enzyme was measured at varying either substrate, using NADPH (panel C) or NADH (panel D) as the electron donor. Reported data are mean ± SE over three technical replicates. The whole analysis was repeated with three different enzyme preparations, and similar data were obtained. Both Michaelis-Menten constants and *V*_MAX_ values pointed at a higher catalytic efficiency with NADH.

In order to investigate whether some aminomethylenebisphosphonic acids that had been previously identified as effective inhibitors of plant^28–31^ and bacterial^32^ P5C reductase have the potential to inhibit also the human enzyme, P5C reductase-1 was purified through heterologous expression in *Escherichia coli* (Figure 2(A,B)). A stepwise elution protocol with increasing imidazole concentrations allowed resolving the enzyme, with an expected denatured molecular mass of 34.2 kDa, from most contaminating proteins. Only another minor band with a denatured mass of about 70 kDa was evident upon SDS-PAGE analysis, which could be a dimer of P5C reductase itself, as found in other cases^39–41^. Mean specific activity of the homogeneous enzyme was 2893 ± 138 nkat mg^−1^ protein when assayed using NADPH as the electron donor. Activity was not perfectly stable, with a half-life of about 2 weeks at 4 °C (data not shown), yet the amount of protein attainable from a single preparation from 400 mL of induced *E. coli* cells allowed to carry out several thousand assays. A kinetic characterisation of the enzyme was performed to define proper conditions for activity assay (Figure 2(C,D)). Consistently with previous results^42–44^, data showed a lower *K_M_* and a higher *V*_MAX_ for NADH. High *K_M_* values for P5C hampered the attainment of completely saturating conditions, especially when NADPH was the co-factor.

**Figure 3.**
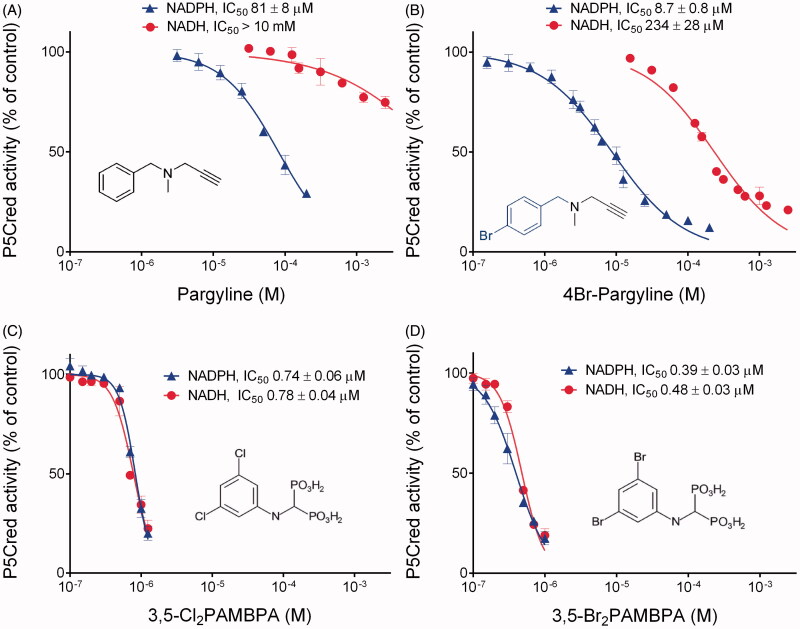
Inhibition of human P5C reductase 1 by pargylines or aminomethylene-bisphosphonates. The purified enzyme was assayed in the presence of increasing concentrations of each compound, as indicated. Parallel controls were carried out in the absence of any inhibitor. Mean activity value in control samples was set as 100%, and activity in treated samples was expressed as percent of such value. Percent values were plotted against the logarithm of inhibitor concentration. Non linear regression of data allowed the calculation of the concentrations causing 50%-inhibition of enzyme activity (IC_50_). In the case of pargylines (panels A and B), different results were obtained using NADPH or NADH as the electron donor, whereas with aminomethylenebisphosphonic acids (panels C and D) almost overlapping curves were evident. Reported results are mean ± SE over three technical replicates. The whole experiment was repeated twice with different enzyme preparations, and consistent patterns were obtained.

Under these optimised experimental conditions, the effect of increasing concentrations of some previously-described inhibitors of human P5C reductase^27^ was at first assessed. With NADPH as the electron donor, pargyline and 4-bromopargyline confirmed their efficacy (Figure 3(A,B)), with IC_50_ values (81 and 9 μM, respectively) that were compatible with those (198 and 9 μM) found hitherto^27^. A strikingly lower effectiveness was on the contrary evident if NADH was used instead, with IC_50_ values more than one order of magnitude higher (Figure 3(A,B)). When the same experiment was performed with phenyl-substituted aminomethylenebisphosphonates, a remarkably different picture was obtained. Both the 3.5-dichlorophenyl- and the 3.5-dibromophenyl-derivative almost completely suppressed the activity of human P5C reductase at concentrations as low as 1 μM, and very similar IC_50_ values were found irrespectively of the pyridine dinucleotide used (Figure 3(C,D)). The effect of some other analogues with a different substitution pattern in the phenyl ring^32^ was also determined. Although several of them were found active in the micromolar range (data not presented), no one showed higher effectiveness than Br_2_PAMBPA, which was therefore used for further determinations.

### Kinetic analysis of the inhibition brought about by Br_2_PAMBPA on human P5C reductase 1

**Figure 4.**
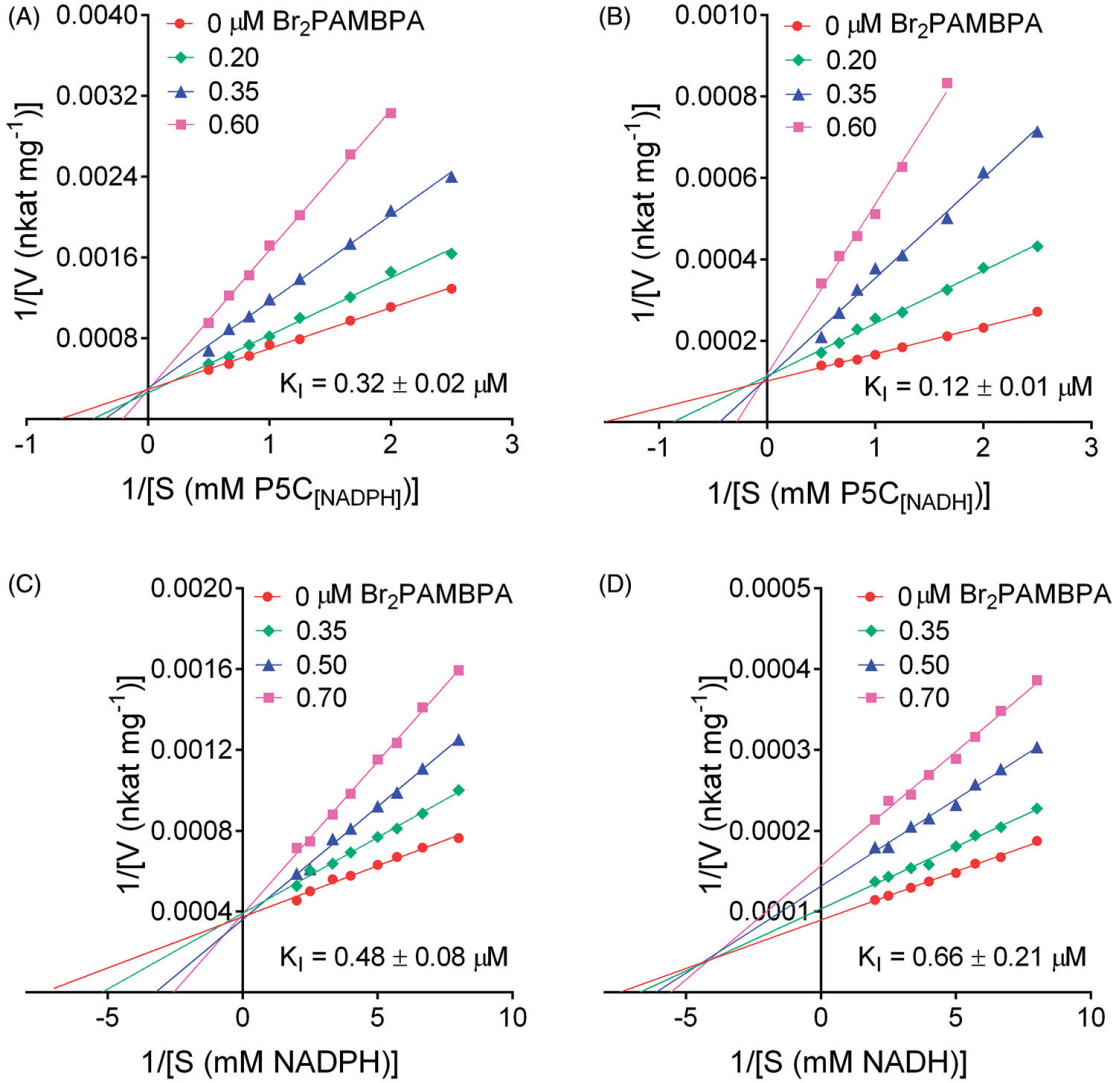
Kinetic analysis of the inhibition of *Hs*PYCR1 by Br_2_PAMBPA. The catalytic rate of the purified enzyme was measured at varying the concentration of a given substrate in the absence or in the presence of increasing levels of the inhibitor. Lines converging to the *Y*-axis in double reciprocal (Lineweaver-Burk) plots accounted for an inhibition of competitive type against P5C both when using NADPH (panel A) or NADH (panel B) as the electron donor. The same result was evident with respect to NADPH (panel C). On the contrary, with NADH as the variable substrate the pattern was suggestive of an inhibition of mixed-type (panel D). Data are mean over three technical replicates. The whole analysis was repeated three times, and similar patterns were obtained. The reported K_I_ values are mean ± SD over replications.

Aminomethylenebisphosphonates had been found to inhibit plant^28^ and bacterial^32^ P5C reductases with a mechanism of non competitive type with respect to NAD(P)H and of uncompetitive type with respect to P5C. To ascertain whether the same holds true for human P5C reductase, a kinetic analysis was performed. Results are summarised in Figure 4. With P5C as the variable substrate, lines converging to the Y-axis in Lineweaver-Burk plots were consistent with an inhibition of competitive type with either NADPH (panel A) or NADH (B) as the electron donor. A similar pattern was obtained at varying NADPH concentration (Figure 4(C)), whereas with NADH results were consistent with a mixed type mechanism of inhibition (Figure 4(D)). A mechanism of competitive type may be an unfavourable feature for an inhibitor, since substrate accumulation *in vivo* might lead to a partial reversal of the inhibitory effect. To take one step further, experiments were therefore carried out to verify whether P5C reductase inhibition by Br_2_PAMBPA takes place inside human cells, leading to proline starvation.

### Effect of Br_2_PAMBPA on the growth of human cancer cell lines

**Figure 5.**
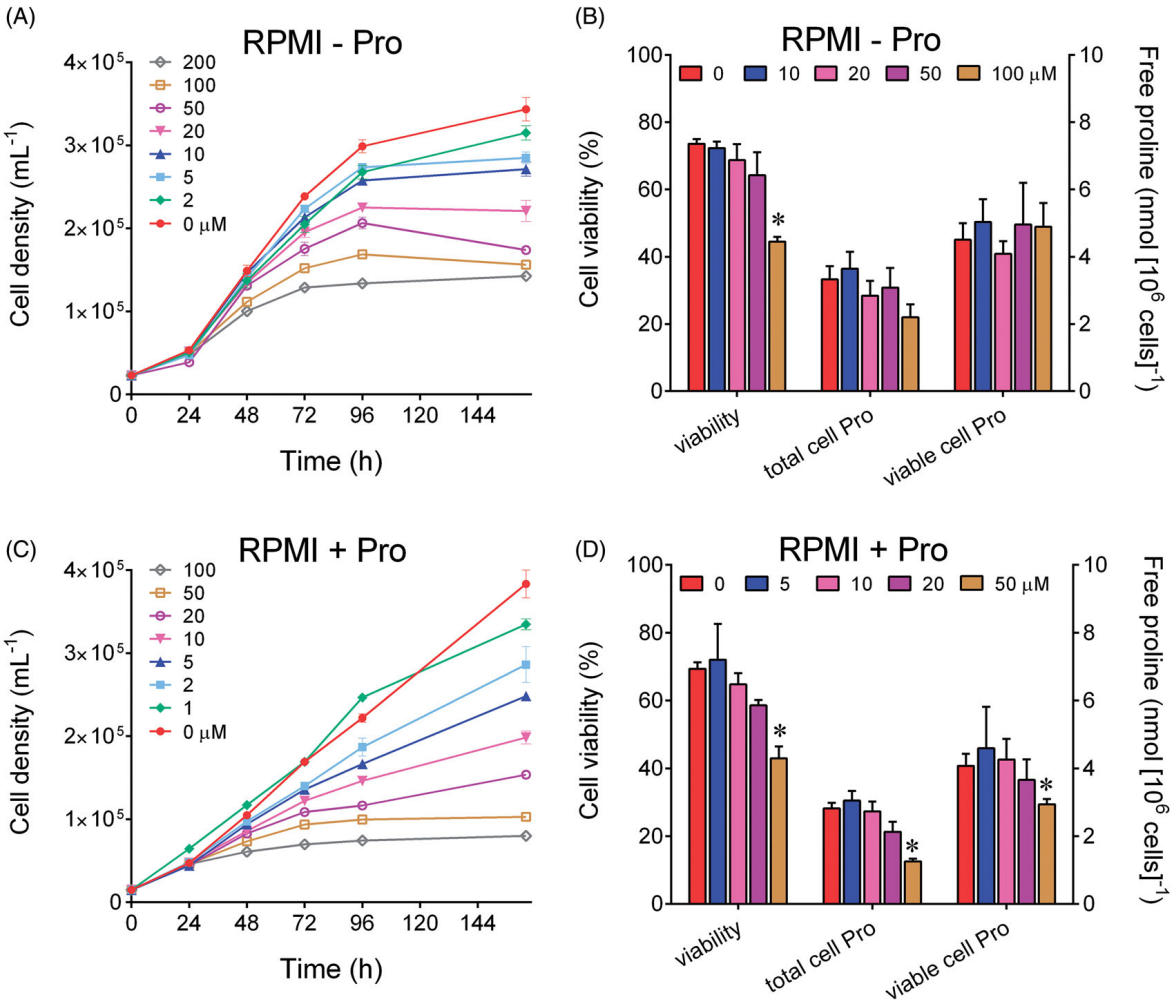
Effect of micromolar levels of Br_2_PAMBPA upon the growth of human myelogenous leukaemia K562 cells. The compound was added 5 h after seeding to a modified RPMI medium not containing proline, and the resulting growth was followed until untreated controls reached the early stationary phase (panel A). Cell viability and free proline content were measured by destructive harvest in parallel samples 96 h after the inoculum. Proline concentration was expressed with respect to either total or viable cells (panel B). The same set of determinations was carried out also in unmodified RPMI medium containing 20 mg L^−1^ Pro (panels C and D). In all cases results are means ± SD over biological triplicates. Statistical significance of differences between treated and control samples in panels B and D were determined by multiple *t* test using the Holm-Sidak method, with *α* = 1.00% (*).

When increasing concentrations of Br_2_PAMBPA were added to a modified RPMI medium not containing proline, the growth of human myelogenous leukaemia K562 cells was indeed found to be inhibited in the range from 2 to 200 μM, with an effect that was proportional to the dose. Inhibition took place 24–36 h following the addition, and 50%-inhibition was achieved at about 20 μM. Interestingly, not only the growth rate was slowed down, but treated cells reached a plateau at a lower cell density than untreated controls (Figure 5(A)). Cell viability was significantly reduced at concentrations exceeding 50 µM, yet most cells were still alive at doses at which growth had been severely impaired (Figure 5(B)). Remarkably, when the intracellular levels of free proline were quantified, concentrations in treated cultures were not significantly different than in controls (Figure 5(B)), suggesting that the inhibitory effect was not related to proline starvation. Consistently, when the experiment was repeated in unmodified RPMI medium containing 20 mg L^−1^ (174 μM) Pro, the inhibition was not reversed. On the contrary, a slightly higher effect was evident, with 50% inhibition at about 7 μM (Figure 5(C)), and a more pronounced effect on cell viability (Figure 5(D)). Once again, when free proline content was expressed with respect to viable cells, no reduction was evident up to 50 µM, dose at which less than 50% reduction was evident in cells whose proliferation has been almost completely abolished. In all cases, no detectable (> 1 nmol [10^6^ cells]^−1^) levels of P5C were found.

**Figure 6.**
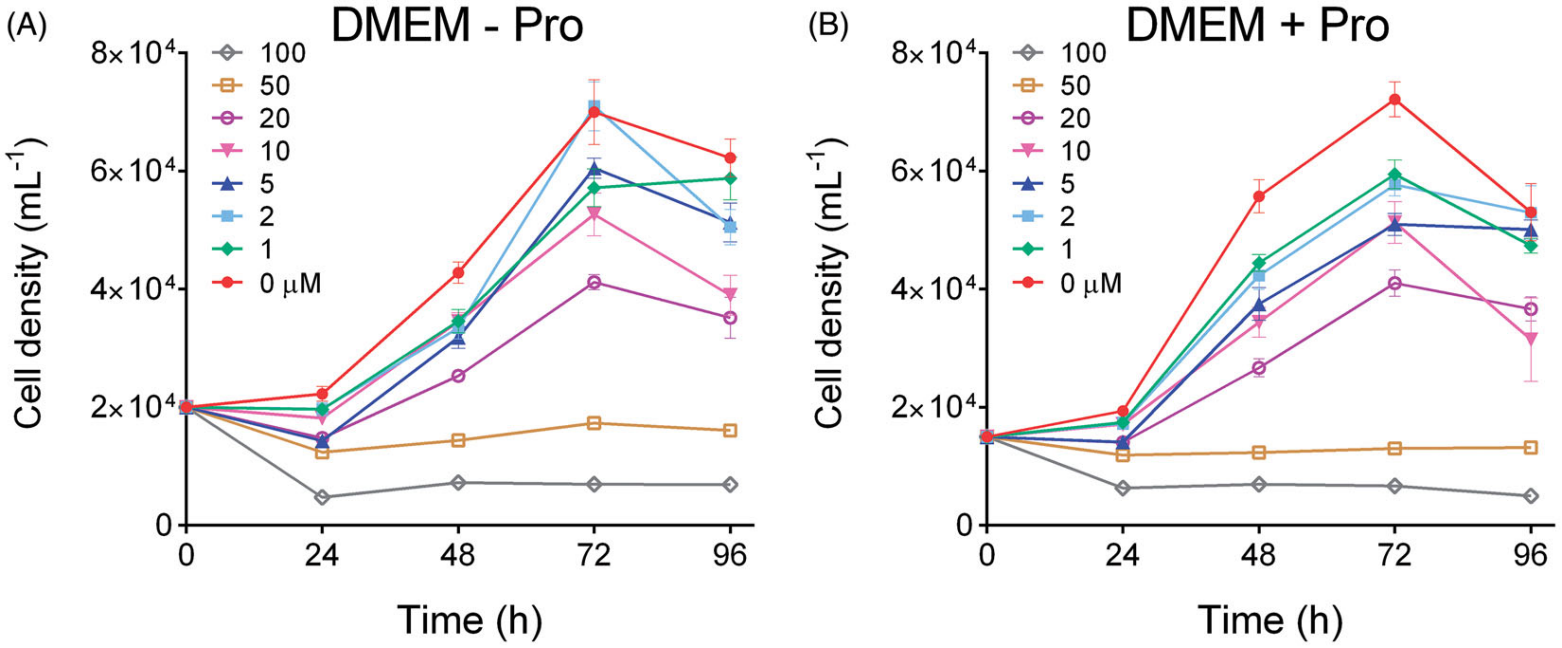
Effect of micromolar levels of Br_2_PAMBPA upon the growth of human epithelial breast cancer MDA-MB-231 cells. The compound was added to DMEM medium 5 h after seeding, and 2% FBS was added 24 h thereafter. The resulting growth was followed by destructive harvest until untreated controls reached confluence (panel A). The same experiment was carried out also in DMEM medium supplemented with 20 mg L^−1^ Pro (panel B). In all cases results are means ± SE over six biological replicates.

To obtain further evidence, similar experiments were carried out with a breast cancer cell line (MDA-MB-231) in which the inhibition of P5C reductase 1 by small-hairpin RNA had been reported to significantly reduce growth and invasion capabilities^45^. However, in this case cell proliferation strictly required the addition of FBS to DMEM medium, and preliminary experiments carried out in standard medium showed only mild effects at Br_2_PAMBPA concentrations exceeding 50 μM (data not shown). Because such a failure may depend on the interaction between the inhibitor and some FBS components, preventing (or greatly reducing) its uptake by human cells^46^, cell cultures were treated with the bisphosphonate under serum-free conditions, and FBS was added 24 h thereafter. By using this protocol, confirmative results were obtained (Figure 6). Starting from 5 μM, both cell growth rate and maximal cell density were progressively reduced, and from 50 μM onward cell proliferation was completely abolished (Figure 6(A)). Almost identical patterns were obtained when the medium was supplemented with 20 mg L^−1^ Pro, condition under which the growth of untreated controls was slightly speeded up (Figure 6(B)).

## Discussion

In human cells, three forms of P5C reductase are present that have specialised roles and different subcellular localizations^44^. The mitochondria-localized PYCR1 is believed to play a main role in the production of proline from glutamate for protein synthesis. PYCR1 has similar K_M_s for NADH and NADPH, but a higher reaction rate with NADH, and is competitively inhibited by proline. During recent years, PYCR1 has attracted increasing interest because its upregulation has been found involved in tumour growth and metastatic progression of several types of cancers^2–5^. A wide study of mRNA profiles from about two thousand tumours across 19 different cancer types showed PYCR1 as one of the most consistently overexpressed metabolic genes^47^, and its downregulation was found to reduce tumour growth and invasion capabilities, making cancer cells more susceptible to conventional chemotherapeutics^45^. Therefore, P5C reductase represents nowadays an attractive potential target for cancer therapy.

Preliminary results towards the identification of specific inhibitors for human P5C reductase have been recently described, showing 4-bromopargyline as the most effective substance^27^. However, P5C reductase had been previously exploited as a potential target for herbicides. A long lasting research in our laboratory allowed identifying halogen-substituted derivatives of phenyl-aminomethylene-bisphosphonic acid as potent inhibitors of the plant enzyme^28–31^. The same compounds were also found to efficiently inhibit the enzyme from a bacterial pathogen^32^. Their evaluation on human P5C reductase *in vitro*, herein reported, showed an even higher effectiveness, with IC_50_ values two orders of magnitude lower than those for the plant enzyme, and tenfold lower than that for 4-bromopargyline. Moreover, the same inhibition patterns were found using either NADH or NADPH as the electron donor, whereas in our hands pargylines showed efficacy in the micromolar range only against the NADPH-dependent activity. The higher sensitivity of human P5C reductase corresponded to a different kinetic mechanism. A remarkable difference between the amino acid sequence of the mammal and the plant enzyme does indeed exist, resulting into significant variations in their three-dimensional structure^6^^,^^41^ that seem thus to involve the binding site for aminobisphosphonates and influence their interaction. A mechanism of competitive type may represent a drawback, in that substrate accumulation deriving from enzyme inhibition could relieve at least in part the effect of the inhibitors *in vivo*. Confirmative results in a cellular system are thus crucial to support the possibility of using aminomethylene-bisphosphonates to reduce P5C reductase activity inside the cell.

When increasing concentrations of the most active compound were added to the culture medium of two human cancer cell lines, antiproliferative effects were in fact found starting from levels about tenfold higher than IC_50_ values *in vitro*. This notwithstanding, cell growth was progressively reduced in the range from 2 to 20 µM Br_2_PAMBPA, and completely abolished at higher concentrations. Interestingly, even at levels at which proliferation was only slowed down, cell cultures did not attain the same maximal densities than untreated controls, but arrested growth at values that were inversely proportional to the dose. Results obtained with myelogenous leukaemia K562 cells, which grow in suspension to highest densities^36^, were confirmed with substrate-adhering breast cancer MDA-MB-231 cells that were chosen as a validated system in which PYCR1 is overexpressed and its inhibition causes the reduction of growth and invasion capabilities^45^. In the latter case, the addition of FBS to the medium was found to greatly reduce Br_2_PAMBPA effectiveness, most likely as a consequence of inhibitor binding to BSA or other serum components^46^, which may prevent the bisphosphonate from entering the cell. This would represent a serious obstacle for *in vivo* treatments with the compound, yet the problem could be overcome by the adoption of suitable delivery systems^48^.

Interestingly, neither Br_2_PAMBPA treatment led to proline starvation, nor the exogenous supply of the amino acid decreased its effectiveness. These results might be consistent with the occurrence of other unrelated target(s) of bisphosphonates in cell metabolism. In fact, the same compounds were found in plants to inhibit also the activity of both cytosolic and plastidal forms of glutamine synthetase, with similar or even higher effectiveness than P5C reductase^49–50^. However, the possibility that the observed antiproliferative effects may depend on the inhibition of glutamine synthetase is unlikely, since the human enzyme has been found substantially insensitive to these compounds (IC_50_ for 3.5-dichlorophenyl-AMPBA of 780 ± 30 µM)^51^. Moreover, also phytotoxic effects of these bisphosphonate were found unrelated to proline starvation, which in treated tissues on the contrary accumulated along with the putatively toxic intermediate P5C^31^. Both in control and treated K562 cells P5C was undetectable, and free proline concentration was constant in the range 4–5 nmol (10^6^ cells)^−1^, levels that were found conserved independently of the presence of exogenous proline in the culture medium, suggesting a strict homeostatic control^52^. Only the treatment with highest Br_2_PAMBPA concentrations caused a slight but significant lowering in free proline content. Such results are consistent with those obtained with 4-bromopargyline, whose effect on the intracellular level of proline was not proportional to the dose^27^.

On the whole, data strongly suggest that the antiproliferative activity of P5C reductase inhibitors is mediated by an interference with the proline-P5C cycle. In this half-biosynthetic, half-catabolic set of reactions, proline is oxidised by ProDH to P5C but the latter, instead of being further oxidised to glutamate in the mitochondrion by P5CDH, is reduced back to proline in the cytosol by P5C reductase (Figure 1). Without resulting in a net change of free proline level, this apparently futile cycle may allow the transfer of reducing equivalents from the cytosol to the mitochondrion, fuelling the respiratory chain^11–13^. An increased activity of this cycle can improve cancer cell survival, proliferation, and metastasis through a variety of mechanisms ranging from enhanced ATP production, nucleotide synthesis, anaplerosis, and redox homeostasis^24^. Inhibition of P5C reductase is therefore expected to interfere with all these mechanisms, hampering cell growth even without impacting on proline availability for protein synthesis.

Further information is obviously required to strengthen the possibility of using Br_2_PAMBPA or other bisphosphonates for cancer therapy. Next steps will be a deeper insight on the molecular mechanisms fostering cell growth inhibition, the study of the effects against non tumoral cells, and the evaluation of the therapeutic potential in more sophisticated experimental systems. In any case, the availability of specific and effective inhibitors of P5C reductase could help elucidating the functions of the P5C-proline cycle in either physiological or pathological environments. Work is currently in progress with these aims in our laboratory.

## Supplementary Material

Supplemental Material
