# Supplementary material for: Phenyl-substituted aminomethylene-bisphosphonates inhibit human P5C reductase and show antiproliferative activity against proline-hyperproducing tumour cells
Source: J Enzyme Inhib Med Chem. 2021 Jun 9;36(1):1248–57. doi: 10.1080/14756366.2021.1919890 (PMC8205077; doi:10.1080/14756366.2021.1919890)
Supplement: Supplemental Material [file IENZ_A_1919890_SM6990.pdf]

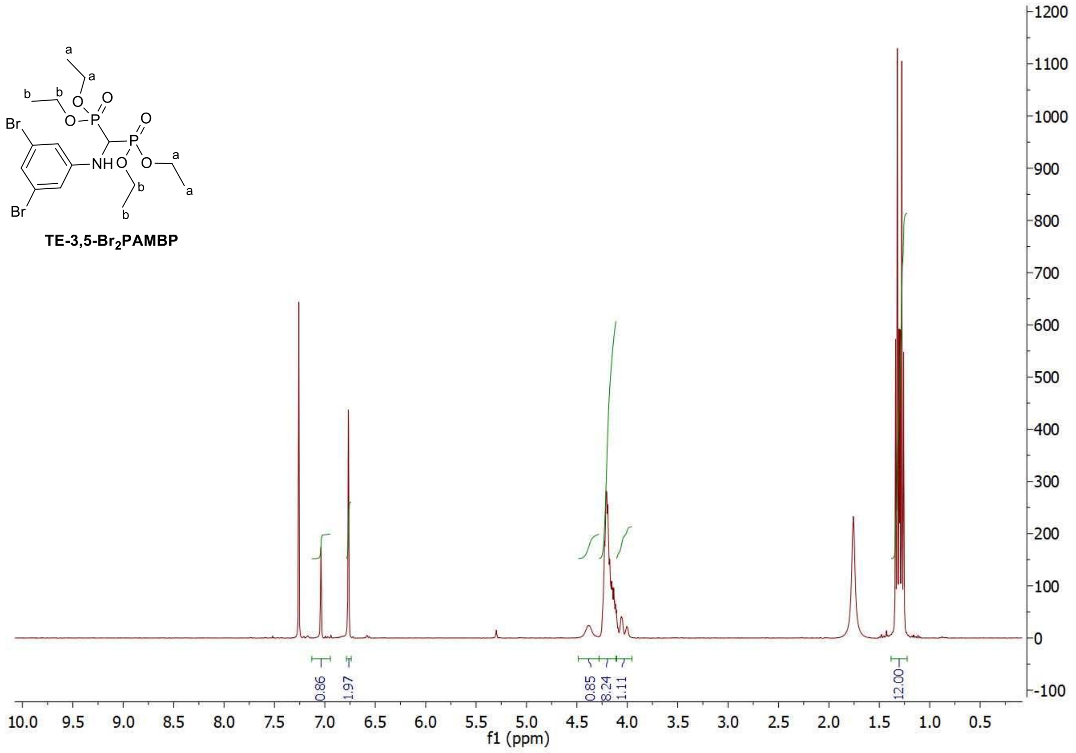

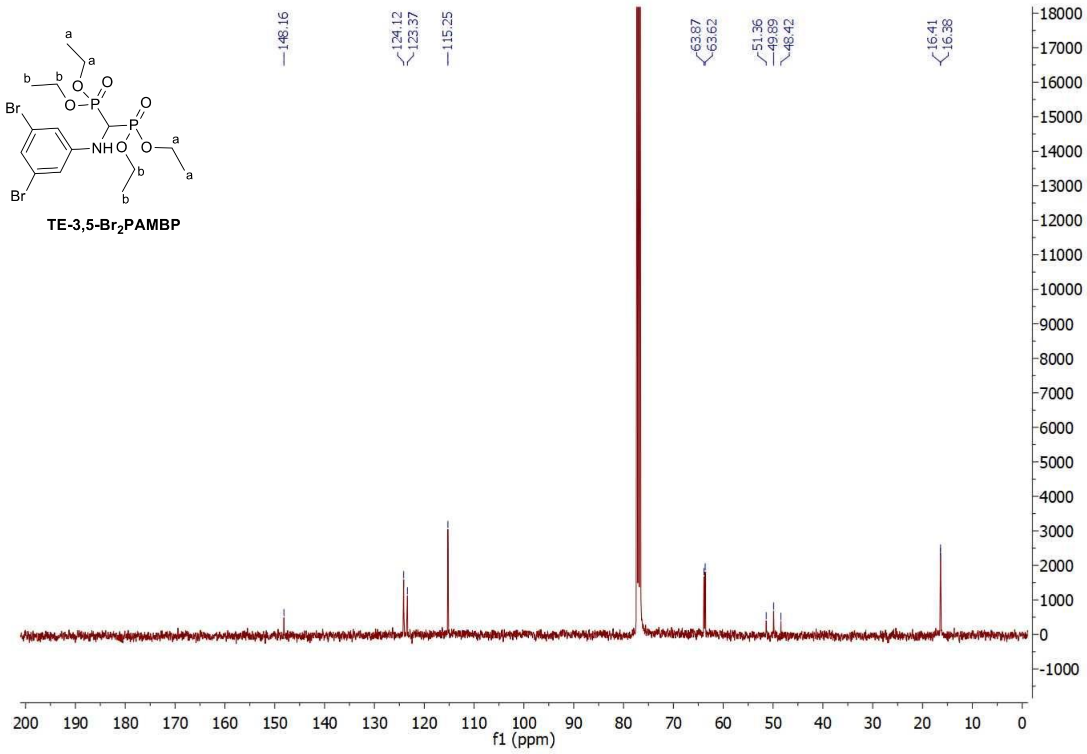

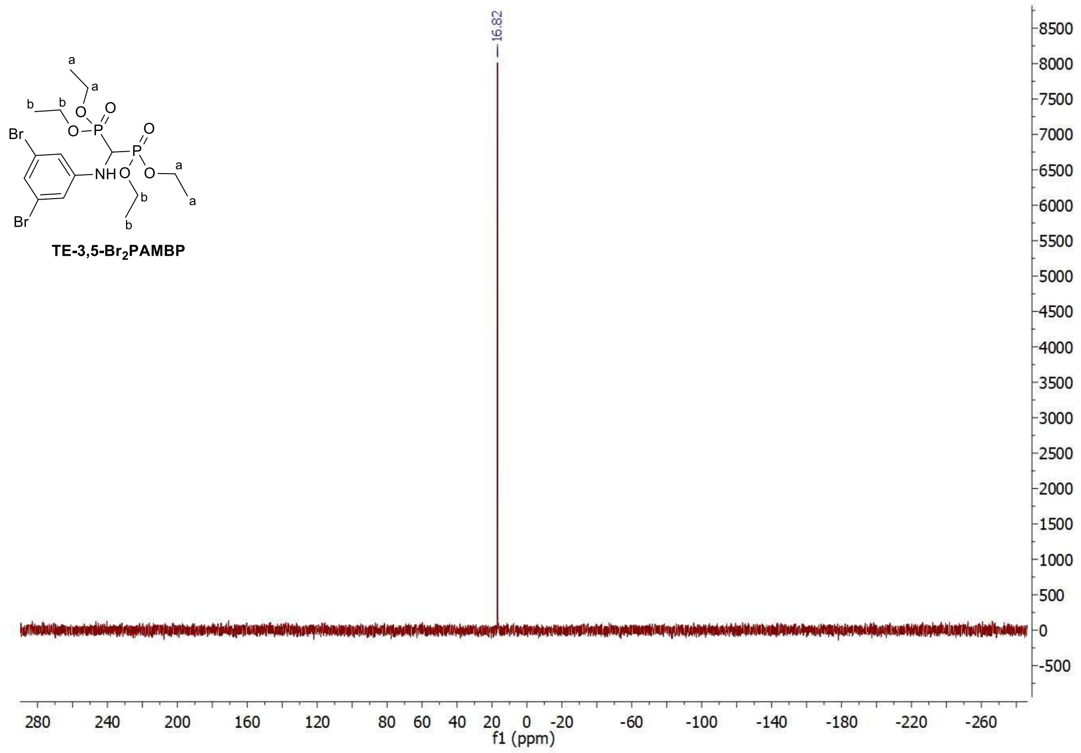

Supplementary Figure S1. ^{1}H (300 MHz), ^{13}C (101 MHz), and ^{31}P (122 MHz) spectra ($CDCl_3$) of tetraethyl (3,5-dibromophenylaminomethylene)bisphosphonate. The chemical shifts in ^{1}H and ^{13}C NMR spectra were referenced to trimethylsilane (TMS), while in ^{31}P NMR were referenced to 85% H_3PO_4 in D_2O. Peak assignments were aided by ^{1}H-^{1}H COSY and gradient-HMQC experiments.

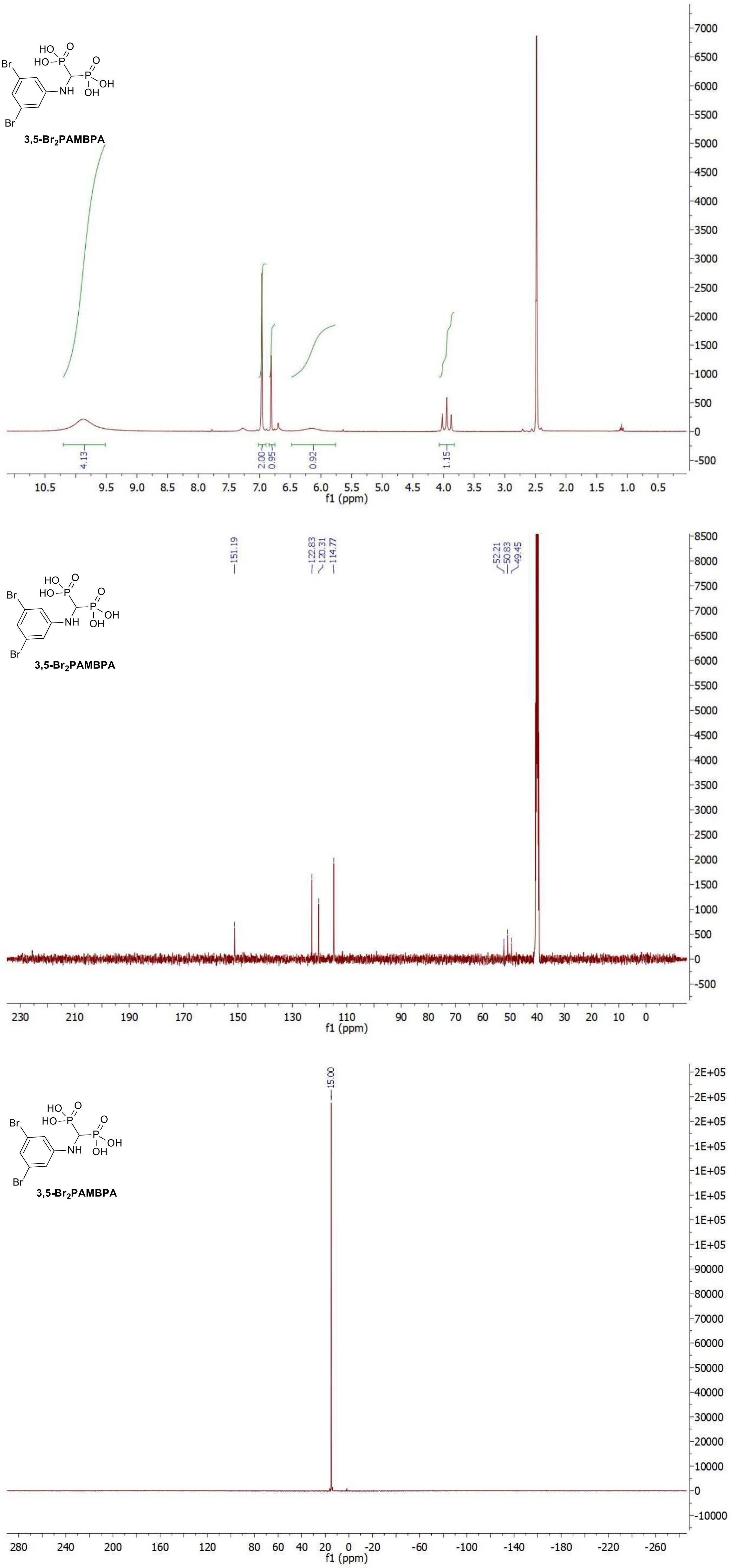

Supplementary Figure S2. ^{1}H (300 MHz), ^{13}C (101 MHz), and ^{31}P (122 MHz) spectra (DMSO-*d*$_6$) of 3,5-dibromophenylaminomethylene-bisphosphonate. The chemical shifts in ^{1}H and ^{13}C NMR spectra were referenced to trimethylsilane (TMS), while in ^{31}P NMR were referenced to 85% H_3PO_4 in D_2O. Peak assignments were aided by ^{1}H-^{1}H COSY and gradient-HMQC experiments.
